# Association of pericardial adipose tissue with left ventricular structure and function: a region‐specific effect?

**DOI:** 10.1186/s12933-021-01219-4

**Published:** 2021-01-25

**Authors:** Jin-Seok Kim, Seon Won Kim, Jong Seok Lee, Seung Ku Lee, Robert Abbott, Ki Yeol Lee, Hong Euy Lim, Ki-Chul Sung, Goo-Yeong Cho, Kwang Kon Koh, Sun H. Kim, Chol Shin, Seong Hwan Kim

**Affiliations:** 1 Division of Cardiology, Korea University Ansan Hospital, 123, Jeokgeum-ro, Danwon-gu, Gyeonggi-do 15355 Ansan, South Korea; 2 Institute of Human Genomic Study, Korea University Ansan Hospital, 123, Jeokgeum-ro, Danwon-gu, Gyeonggi-do 15355 Ansan, South Korea; 3 Division of Radiology, Korea University Ansan Hospital, Ansan, Korea; 4 Division of Cardiology, Hallym University Sacred Heart Hospital, Anyang, Korea; 5 Division of Cardiology, Kangbuk Samsung Medical Center, Seoul, Korea; 6 Division of Cardiology, Seoul National University Bundang Hospital, Seongnam, Korea; 7 Division of Cardiology, Gachon University Gil Medical Center, Incheon, Korea; 8 Division of Endocrinology, Gerontology and Metabolism, Stanford Diabetes Research Center, Stanford University School of Medicine, Stanford, CA USA

**Keywords:** Pericardium, Adipose tissue, Tissue doppler echocardiography, Left ventricular function, Left ventricular hypertrophy

## Abstract

**Background:**

The independent role of pericardial adipose tissue (PAT) as an ectopic fat associated with cardiovascular disease (CVD) remains controversial. This study aimed to determine whether PAT is associated with left ventricular (LV) structure and function independent of other markers of general obesity.

**Methods:**

We studied 2471 participants (50.9 % women) without known CVD from the Korean Genome Epidemiology Study, who underwent 2D-echocardiography with tissue Doppler imaging (TDI) and computed tomography measurement for PAT.

**Results:**

Study participants with more PAT were more likely to be men and had higher cardiometabolic indices, including blood pressure, glucose, and cholesterol levels (all *P* < 0.001). Greater pericardial fat levels across quartiles of PAT were associated with increased LV mass index and left atrial volume index (all *P* < 0.001) and decreased systolic (*P* = 0.015) and early diastolic (*P* < 0.001) TDI velocities, except for LV ejection fraction. These associations remained after a multivariable-adjusted model for traditional CV risk factors and persisted even after additional adjustment for general adiposity measures, such as waist circumference and body mass index. PAT was also the only obesity index independently associated with systolic TDI velocity (*P* < 0.001).

**Conclusions:**

PAT was associated with subclinical LV structural and functional deterioration, and these associations were independent of and stronger than with general and abdominal obesity measures.

## Background

Pericardial adipose tissue (PAT), which refers to the combination of epicardial adipose tissue (EAT, fat enclosed by the pericardial sac) and paracardial fat (fat surrounding the external surface of the pericardium), is associated with the development of cardiovascular disease (CVD) and events [1]. In particular, increased EAT has been known to be associated with atrial fibrillation [2], coronary artery calcification [3], impaired left atrial (LA) and left ventricular (LV) structure and function in various clinical settings [4–7]. Because of its anatomic contiguity to the myocardium and the coronary arteries, EAT seems to mediate adverse cardiac effects by direct lipotoxicity, local compressive forces, and/or endocrine/paracrine effects [8]. Likewise, there are some data which show the association of PAT with atrial fibrillation and LV remodeling [1, 9]. However, given that epicardial or paracardial fat accumulation is just among a variety of ectopic fat depots (e.g., in liver, pancreas, and muscle) associated with general obesity, there is some controversy regarding its independent role in heart diseases beyond those associated with the standard indices of excess adiposity, such as body mass index (BMI) and waist circumference (WC). Indeed, the Framingham Heart Study has demonstrated significant associations between EAT and paracardial fat with cardiac structure and function, but these associations did not persist after adjustment of body weight, suggesting that the systemic effects of obesity outweigh local effects of EAT and paracardial fat [10]. On the other hand, other studies have shown independent associations between EAT and LV mass, LA size, as well as LV systolic or diastolic function [11–14]. These recent studies, however, were limited by their small sample size and lack of adjustment for general adiposity measures. Most of all, because they have focused on only EAT, disregarding the potential contribution of paracardial fat on LV structure and function, little is known whether the PAT, including paracardial fat, is associated with the changes of LV structure and function, independent of general adiposity measures, despite its correlation with CVD.

In this study, we investigated the relationship of PAT with LV structure and function using the tissue Doppler imaging technique after accounting for clinical measures of general obesity in a large community-based cohort study.

## Methods

### Study population

The study population was recruited from an ongoing population-based Ansan cohort embedded in the Korean Genome Epidemiology Study, which is described in detail elsewhere [15]. Briefly, the baseline cohort comprised of 5020 participants and has been followed biennially from 2001. This eighth follow-up examination was conducted between March 2015 and December 2016. A total of 3083 Ansan cohort participants were invited to participate in a substudy for a more extensive CV evaluation, including chest computed tomography (CT) and 2D transthoracic echocardiography. Among these individuals, a total of 2524 completed conventional and tissue Doppler echocardiography and chest CT for the measurement of PAT. From this sample, we excluded individuals who had incomplete echocardiography data (n = 7) or CT data (n = 2); known CVD, including a previous history of myocardial infarction, congestive heart failure, congenital heart disease, coronary revascularization, angina, stroke, cardiomyopathy, significant valvular heart disease, arrhythmia, pericardial effusion, and LV ejection fraction < 50 % (n = 45); or a serum creatinine level ≥ 2.0 mg/dL (n = 7), leaving a total of 2471 subjects for the analysis.

The protocol of the study was approved by the Human Subjects Review Committee at the Korea University Ansan Hospital, and all participants provided written informed consent at every site visit.

### Echocardiography

Standard 2D echocardiography examinations were performed by an expert using the Vivid 7 system (GE Vingmed, Horton, Norway) with a 4-MHz transducer. Cardiac chamber diameters and wall thickness were measured according to the current recommendations from the American Society of Echocardiography and the European Association of Cardiovascular Imaging [16]. The LA volume and LV mass were calculated using the area-length method and Devereux formula and were indexed to body surface area. LV ejection fraction measurement was conducted using the modified biplane Simpson’s method. The peak transmitral E and A diastolic velocities and the deceleration time (DT) were recorded in the apical 4-chamber view at the tips of the mitral valve leaflets during diastole. The systolic tissue Doppler imaging (TDI) Sm and early diastolic Em velocities were measured at the septal side of the mitral annulus. Subsequently, the mitral E/Em ratio was calculated as an index of LV diastolic filling pressure. Echocardiographic LV hypertrophy was defined as an LV mass index > 95 g/m^2^ for women and > 115 g/m^2^ for men. Subclinical LV diastolic dysfunction was defined if at least two of the following conditions were met: (i) septal TDI Em velocity ≤ 7 cm/s; (ii) septal E/Em ratio > 15; (iii) LA volume index ≥ 34 mL/m^2^ [17].

### Pericardial fat volume quantification

**Fig. 1:**
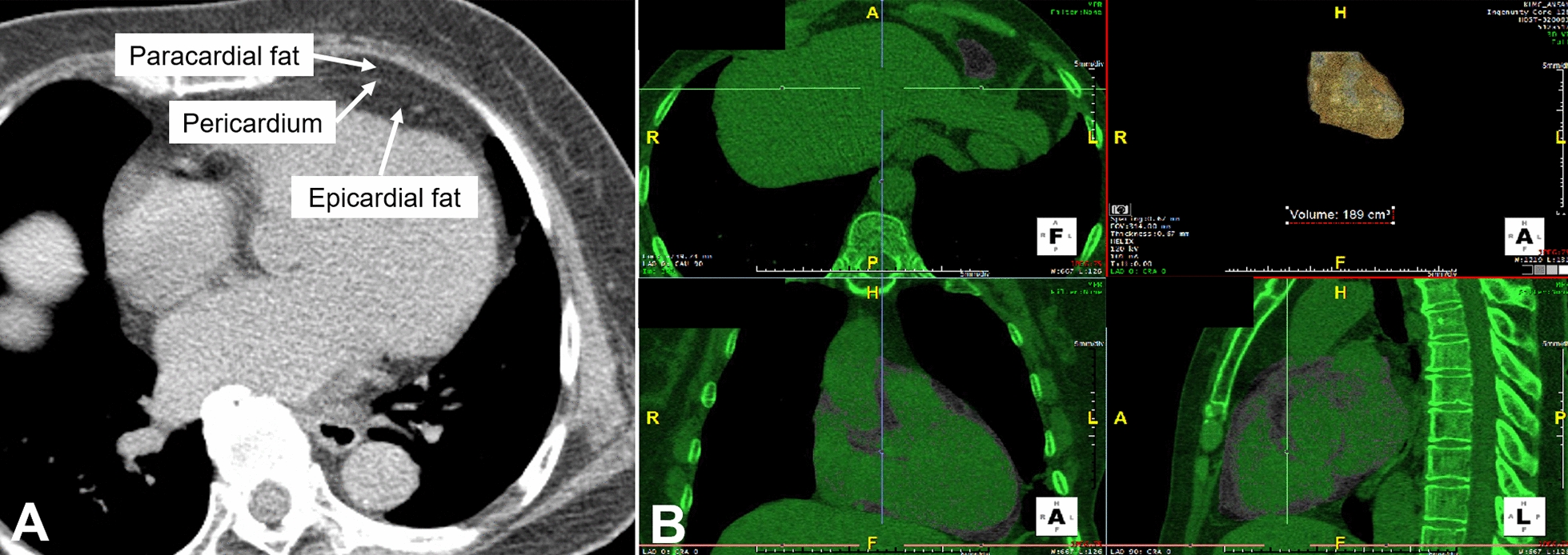
Definition of pericardial adipose tissue (epicardial adipose tissue plus paracardial adipose tissue) in an axial image (**a**). Gray overlay represents measurement of pericardial fat (epicardial fat and paracardial fat) (**b**). For this subject, pericardial fat was 189 cm^3 ^

**Fig. 2:**
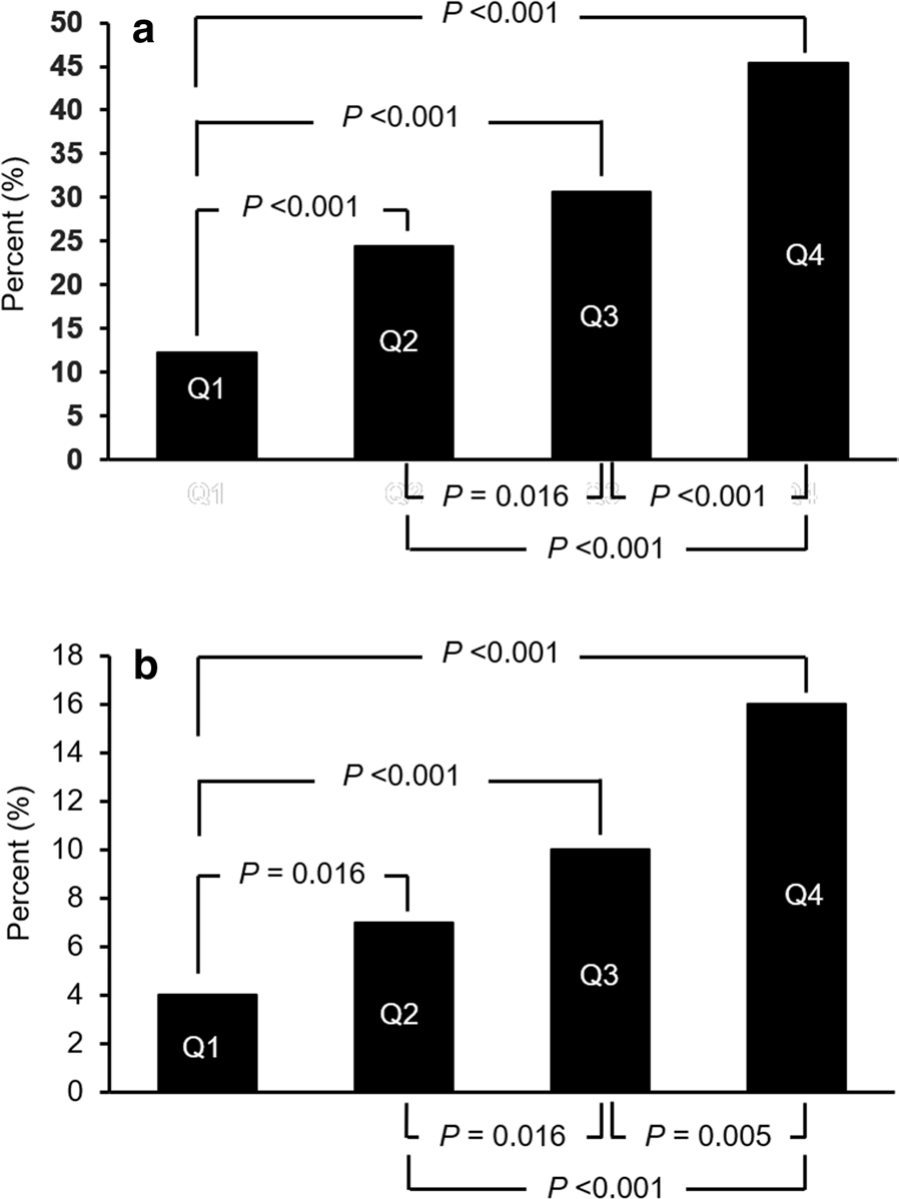
The prevalence of LV hypertrophy (**a**) and LV diastolic dysfunction (**b**) in the quartiles of pericardial adipose tissue

Details on the procedures for chest CT data acquisition, scan quality assurance, and scan reading have been described previously [18]. Briefly, participants underwent chest CT scans by well-trained technicians using a commercial 64-multidetector CT (Brilliance 64, Philips Healthcare, Cleveland, OH, USA) according to a standardized protocol. CT images were acquired in the supine position during end-inspiratory and end-expiratory breath holds. Scanning parameters were held constant at the 64 × 0.625 mm detector configuration, 120 kV (peak), 100 mAs, and a section thickness of 0.625 mm without intravenous contrast material. Pericardial fat measurements were performed three-dimensionally using Aquarius iNtuition Edition software version 4.4.11 (TeraRecon Headquarters, Foster City, CA, USA), which is an automated lung image analysis tool, with non-contrast chest CT images (Fig. 2). PAT included adipose fat located in the pericardium and in the thorax from the level of the pulmonary artery bifurcation to the diaphragm (superior or inferior) and from the chest wall to the descending thoracic aorta (anterior or posterior). Segmentation of the overall volume was automatically interpolated using manually defined tracings, and PAT volume for every 1.0 mm was subsequently quantified by calculating the total volume of the tissue in which CT density ranged from − 500 to 0 Hounsfield units within the thoracic cavity. Two experienced radiographers, blinded to clinical information, quantified PAT volume. A random sample of 30 participants was selected, and Spearman correlation of coefficients between-reader and within-reader ranged from 0.92 to 0.97 (Fig. 1).

### Risk factor assessment

Clinical information, CV risk factors, and medical history were obtained using interviewer-administered questionnaires. WC was measured at the level of umbilicus. BMI (kg/m^2^) was defined as body weight (kg) divided by the square of height (m). BMI ≥ 23 kg/m^2^ was classified as overweight, and BMI ≥ 25 kg/m^2^ was classified as obese. Blood pressure was measured according to a standardized protocol using a mercury sphygmomanometer. Hypertension was defined as a systolic blood pressure ≥ 140 mmHg or diastolic blood pressure ≥ 90 mmHg and/or the use of antihypertensive drugs. After an overnight fasting of at least 8 hours, blood samples were collected for serum total cholesterol, high-density lipoprotein (HDL) cholesterol, triglycerides (TG), plasma glucose, HbA1c, and serum creatinine analysis. Type 2 diabetes (T2D) was defined as a fasting blood glucose ≥ 126 mg/dL or the use of either insulin or hypoglycemic agents.

### Statistical analyses

Average (± standard deviation [SD]) and percent study characteristics are presented across quartiles of PAT. Quartiles included the following PAT ranges (cm^3^): Q1 (101–205); Q2 (206–245); Q3 (246–291); Q4 (292–565). We used χ^2^ test for dichotomous variables and analysis of variance (ANOVA) for continuous variables to compare the four groups of PAT. Average (± SD) measures of LV structure and function were also summarized across the quartiles of PAT and were compared using ANOVA. Linear regression modeling was used to assess associations of the adiposity measures as the independent variable with LV structural and functional parameters as the dependent variables. The standardized β coefficient provides the change in measures of LV structure and function per 1 SD increase in the adiposity variable. In addition to the multivariate linear regression model (model 1) adjusting for age, sex, systolic blood pressure, heart rate, serum creatinine, fasting glucose, hypertension treatment, diabetes treatment, smoking, alcohol, and exercise, model 2 added all three adiposity measures (WC, BMI, and PAT). Adiposity parameters were included as continuous variables in both univariate and multivariate analyses. The multivariate model (model 2) was inspected for multicollinearity by calculating the variance inflation factor because of the high-level correlations among adiposity parameters. All reported p-values were based on two-sided tests of significance using the SPSS statistical software package (IBM SPSS statistic 18.0).

## Results

The baseline demographic, clinical, and biochemical measurements stratified by PAT quartiles are shown in Table 1. The mean age of study population was 62 ± 7 years, and 1,214 (49.1 %) were male. The mean PAT was 253 ± 66 cm^3^. There were consistent increases in WC and BMI across the quartiles of PAT (all *P* < 0.001). For those in the top PAT quartile, the mean WC was more than 15 cm higher than that of participants in the bottom quartile (least PAT). The mean BMI was nearly 5 kg/m^2^ higher. PAT showed strong positive correlations with WC (*r* = 0.66, *P* < 0.001) and BMI (*r* = 0.60, *P* < 0.001). The participants with more PAT were older, more likely to be men, consumed higher alcohol amounts and smoked more, and had comorbidity diseases, including hypertension, T2D, or dyslipidemia (all *P* < 0.001), compared to those in the lowest quartile. However, there was no difference in the level of physical activity (*P* = 0.383).

**Table 1:** Clinical characteristics of study participants (n = 2471) stratified by pericardial adipose tissue quartiles

Variable	Q1 (n = 617)	Q2 (n = 622)	Q3 (n = 615)	Q4 (n = 617)	*P* value
Age (years)	59.9 ± 5.6	61.2 ± 6.2	61.7 ± 6.5	63.34 ± 7.6	< 0.001
Male, n (%)	222 (36)	283 (46)	332 (54)	377 (61)	< 0.001
Waist circumference (cm)	76.2 ± 6.2	82.2 ± 6.1	85.4 ± 6.4	91.3 ± 7.7	< 0.001
Body mass index (kg/m^2^)	22.2 ± 2.2	24.1 ± 2.2	25.2 ± 2.4	26.9 ± 2.9	< 0.001
Systolic blood pressure (mmHg)	112 ± 14	115 ± 14	117 ± 14	120 ± 13	< 0.001
Diastolic blood pressure (mmHg)	72 ± 8	74 ± 9	75 ± 9	76 ± 9	< 0.001
Heart rate (bpm)	62 ± 7	63 ± 7	62 ± 9	62 ± 7	0.691
Hypertension (%)	20	33	41	53	< 0.001
Hypertension treatment (%)	16	27	34	45	< 0.001
Diabetes (%)	9	12	13	22	< 0.001
Diabetes treatment (%)	8	10	13	21	< 0.001
Fasting glucose (mg/dL)	94 ± 17	99 ± 26	100 ± 24	104 ± 25	< 0.001
HbA1c (%)	5.7 ± 0.7	5.9 ± 1.0	5.9 ± 0.7	6.1 ± 1.0	< 0.001
Total cholesterol (mg/dL)	196 ± 36	191 ± 34	190 ± 38	186 ± 36	< 0.001
HDL-cholesterol (mg/dL)	51 ± 13	47 ± 12	45 ± 11	43 ± 10	< 0.001
Triglycerides (mg/dL)	115 ± 64	128 ± 78	142 ± 79	153 ± 87	< 0.001
Creatinine (mg/dL)	0.92 ± 0.16	0.96 ± 0.18	0.99 ± 0.19	1.02 ± 0.19	< 0.001
Current smoker (%)	7	8	12	14	< 0.001
Current alcohol drinker (%)	36	42	47	51	< 0.001
Physical activity (MET-min/week)	175 ± 223	181 ± 230	161 ± 218	180 ± 245	0.383
Pericardial adipose tissue (cm^3^)	176 ± 22	226 ± 12	267 ± 13	342 ± 45	< 0.001

HDL indicates high-density lipoprotein

Table 2 shows the associations of PAT with LV structural and functional parameters across the quartiles of PAT. Participants in the highest quartile of PAT had a higher LA volume index, LV mass index, and E/Em ratio and a lower mitral inflow E/A ratio, systolic TDI velocity, and early diastolic TDI velocity (all *P* < 0.001), compared to those in the lowest quartile. There was no significant difference in LV ejection fraction among the PAT quartiles (*P* = 0.326). Similarly, using the predefined definitions of LVH and LV diastolic dysfunction, the prevalence of LV hypertrophy and LV diastolic dysfunction increased with higher quartiles of PAT (Fig. 2).

**Table 2:** Measurements of left ventricular structure and function using 2D-echocardiography in the pericardial adipose tissue quartiles

Variable	Q1 (n = 617)	Q2 (n = 622)	Q3 (n = 615)	Q4 (n = 617)	*P* value
LA volume (mL)	26.5 ± 4.7	27.7 ± 5.4	28.6 ± 6.1	29.1 ± 6.2	< 0.001
LA volume index (mL/m^2^)	26.5 ± 4.7	27.7 ± 5.4	28.6 ± 6.0	29.1 ± 6.2	< 0.001
Relative wall thickness (cm)	0.42 ± 0.07	0.44 ± 0.07	0.45 ± 0.07	0.46 ± 0.07	< 0.001
LV mass (g)	137 ± 27	157 ± 30	169 ± 31	192 ± 36	< 0.001
LV mass index (g/m^2^)	87 ± 14	95 ± 15	99 ± 15	107 ± 17	< 0.001
LV ejection fraction (%)	64 ± 5	64 ± 4	64 ± 5	65 ± 5	0.326
Mitral inflow velocity					
E, cm/s	0.65 ± 0.13	0.62 ± 0.13	0.61 ± 0.14	0.60 ± 0.14	< 0.001
A, cm/s	0.65 ± 0.14	0.68 ± 0.14	0.70 ± 0.16	0.73 ± 0.16	< 0.001
E/A ratio	1.05 ± 0.29	0.95 ± 0.26	0.90 ± 0.23	0.86 ± 0.24	< 0.001
DT, ms	178 ± 34	184 ± 37	187 ± 40	189 ± 41	< 0.001
Tissue Doppler imaging (TDI)					
TDI Sm velocity (cm/sec)	7.43 ± 1.03	7.34 ± 1.06	7.32 ± 1.13	7.23 ± 1.08	0.015
TDI Em velocity (cm/sec)	7.32 ± 1.48	6.73 ± 1.41	6.46 ± 1.26	6.04 ± 1.27	< 0.001
E/Em ratio	9.1 ± 2.0	9.5 ± 2.4	9.7 ± 2.6	10.2 ± 2.6	< 0.001

*DT* deceleration time,* LA* left atrium,* LV* left ventricle,* TDI* tissue Doppler imaging

Graded relations between the adiposity measures (WC, BMI, and PAT) and LV structural and functional measurements were then explored (Table 3). In the unadjusted model and adjusted regression model (model 1 adjusting for age, sex, systolic blood pressure, heart rate, serum creatinine, fasting glucose, hypertension treatment, diabetes treatment, smoking, alcohol, and exercise), all three adiposity indices were significantly associated with LV mass, LA volume, and TDI Ea velocity (all *P* < 0.001). However, in a fully adjusted model (model 2 adding all of adiposity measures in addition to the multivariate model 1), these statistically significant associations were identified in BMI (*P* < 0.001 for LV mass and LA volume and *P* = 0.009 for TDI Ea velocity) and PAT (all *P* < 0.001), but not WC. On the basis of the standardized β, the magnitude of the associations was higher with PAT than with BMI. Interestingly, a statistical significance between systolic TDI velocity and adiposity measures was only observed in association with PAT (*P* < 0.001) in both univariate and multivariate regression models.

**Table 3:** Multivariable-adjusted linear regression models of association of adiposity measures with LV structure and function

	LV structure and function										
	LV mass			LA volume			TDI Sa			TDI Ea	
	β	*P* value		β	*P* value		β	*P* value		β	*P* value
Waist circumference											
Unadjusted	0.568	< 0.001		0.348	< 0.001		0.025	0.205		− 0.285	< 0.001
Model 1	0.398	< 0.001		0.297	< 0.001		− 0.0373	0.121		− 0.215	< 0.001
Model 2	0.042	0.127		− 0.047	0.202		− 0.012	0.765		− 0.047	0.181
Body mass index											
Unadjusted	0.437	< 0.001		0.340	< 0.001		− 0.018	0.367		− 0.261	< 0.001
Model 1	0.390	< 0.001		0.318	< 0.001		− 0.020	0.319		− 0.206	< 0.001
Model 2	0.179	< 0.001		0.225	< 0.001		0.051	0.179		− 0.085	0.009
Pericardial adipose tissue											
Unadjusted	0.577	< 0.001		0.396	< 0.001		− 0.061	0.002		− 0.332	< 0.001
Model 1	0.444	< 0.001		0.337	< 0.001		− 0.082	< 0.001		− 0.226	< 0.001
Model 2	0.310	< 0.001		0.226	< 0.001		− 0.106	< 0.001		− 0.146	< 0.001

Models constructed with cardiovascular measures as dependent variables and obesity parameters as independent variables. β coefficient is per 1 SD of the obesity parameter. Model 1 is adjusted for age, sex, systolic blood pressure, heart rate, serum creatinine, fasting glucose, hypertension treatment, diabetes treatment, smoking, alcohol, and exercise. Model 2 is adjusted for model 1 plus waist circumference, body mass index, and pericardial adipose tissue as continuous variables.

*LV* left ventricle,* TDI* tissue Doppler imaging

## Discussion

In this large population-based cohort of community-dwelling adults, we found strong associations among PAT, cardiometabolic risk factors, and LV structure and function. Although these relationships with LV structure and function were also observed with general adiposity measures, such as WC and BMI, the magnitude of the associations was strongest with PAT than with WC or BMI. Interestingly, only pericardial fat was associated with subclinical LV systolic function, and the association persisted in a multivariate-adjusted model including WC and BMI. The present findings strongly support the role of a local pathogenic effect of PAT on the structure and function of the heart.

### Association of PAT with CVD

Although heterogeneous and inconsistent terminology, such as cardiac ectopic fat, intra-pericardial fat, extra-pericardial fat, intra-thoracic fat, mediastinal fat, and total thoracic fat, has been used to refer to adipose tissue around the heart by authors in any individual study, PAT is generally defined as EAT plus paracardial fat [2]. In the present study, we used this definition to avoid confusion caused by a significant heterogeneity in the terminology already used when comparing with previously published studies.

EAT and paracardial fat are embryologically and anatomically different. Because of its direct anatomical proximity to the myocardium, EAT seems to be more strongly associated with the development of CVD than abdominal visceral fat [19]. A study by Batal et al., which has shown that peri-atrial fat thickness at the mid-left atrium among various EAT around the LA was closely associated with AF burden, highlights a potential local pathogenic effect of EAT [20]. Similarly, PAT (EAT plus paracardial fat) demonstrated a significant association with incident CVD, independent of general adiposity measures, such as BMI and WC [1, 21]. In patients with T2D, however, only high levels of EAT was independently associated with increased risk of incident CVD [22], suggesting that EAT measure in T2D may be superior as a potential biomarker in CVD risk prediction model. Although EAT seems to play a primary role as a direct disease marker in the development of CVD, the measurement of PAT has several advantages over that of EAT [23]. Generally, because a clear demarcation of the thin pericardium for quantification is technically challenging and time-consuming, especially in lean individuals, quantification of PAT is known to be easier and bring better reproducibility [21, 23]. Despite the fact that high paracardial fat depot is found to be associated with adverse cardiometabolic risk profiles [24], the focus of most of the previous research has been heavily on EAT, but not PAT.

The precise mechanisms by which EAT and paracardial fat contribute to CVD development remain unclear. Both increased EAT and paracardial fat could affect LV diastolic filling and LA dilation by a possible mechanical force [10]. In addition, these two fat depots may exert deleterious effect via a local paracrine pathway because these cardiac fat cells represent a very small proportion of total body fat [19]. In 201 participants undergoing non-contrast CT scans, both EAT and PAT were associated with the presence of coronary calcium [25]. However, considering that EAT lies directly on the myocardium without fascial boundaries, it seems likely that EAT, but not paracardial fat, play a major role in the pathogenesis of LV dysfunction, coronary artery calcification, and atrial fibrillation by acting as a source of paracrine factor that stimulates secretion of myocardial inflammatory markers. The study by Rosito et al. showed that while EAT was associated with coronary artery calcification, paracardial fat was associated with abdominal aorta calcification, which points to the different roles of paracrine modulators between EAT and paracardial fat on vascular calcification [26]. Several recent studies have revealed that FABP 4 expression in EAT was strongly associated with the extent of atherosclerosis in coronary artery disease patients with metabolic syndrome [27], and annexin-A2/fetuin-A signaling in EAT was linked to the pathophysiology of coronary atherosclerotic calcification in elderly patients with coronary artery disease [28]. However, since no association between EAT and coronary atherosclerosis was not observed in patients with type 1 diabetes [29], additional pathophysiologic studies are needed about whether the role of EAT on coronary atherosclerosis may differ depending on the type of DM.

### Association of PAT with chamber measures

Numerous studies have shown that general obesity and abdominal fat are associated with increased LV mass and LA volume. On the other hand, there is limited evidence for the associations of EAT or PAT with LV chamber measures. Some have only shown significant associations in univariate analysis [30]. Others have demonstrated inconsistent results depending on CVD status [31] and obesity [12]. In addition, the Jackson Heart Study reported that LV mass and LA size were independent predictors of EAT only among women [32]. Although paracardial fat *per se* did not show a significant association with LV mass [10], the measurement of PAT was associated with adverse alterations in cardiac structure [1]. In line with previous findings, we show a consistent association between PAT with LV mass and LA volume, regardless of the BMI and gender (data not shown) in a large sample. In addition, the magnitude of association between PAT and LV structure was stronger than with measures of generalized adiposity. Thus, the current findings suggest that the measurement of PAT may be a novel biomarker of cardiac remodeling.

### Association of PAT with LV diastolic function

Although a few studies failed to reveal an independent effect of EAT on LV diastolic function [31], most prior work has shown a consistent association between EAT and LV diastolic parameters, even after adjustment for other markers of adiposity [5, 33]. On the other hand, to our knowledge, there has been just one study on whether paracardial fat *per se* is an independent predictor of LV diastolic function. In a sample of 1,004 subjects, Christensen et al. demonstrated that only high EAT volume was associated with impaired LV diastolic function in patients with T2D, whereas paracardial fat did not show any significant association with LV diastolic function in both patients with and without T2D [4]. Instead, several studies which measured PAT (EAT plus paracardial fat) volume have found that PAT accumulation is also independently associated with LV diastolic dysfunction [1, 34]. Specifically, PAT surrounding the LV was associated more with adverse subclinical alterations in LV diastolic function as compared to RV fat, suggesting a region-specific influence of PAT on LV diastolic function [34]. The present findings are consistent with these studies and indicate that PAT may be a novel marker of LV diastolic dysfunction. However, it was also possible that these findings had been caused primarily by the role of EAT rather than by that of paracardial fat on LV diastolic function. Interestingly, in patients with heart failure with preserved EF, excess EAT was not associated with measures of resting LV diastolic function. However, since increased EAT displayed more profound hemodynamic abnormalities with marked elevations in cardiac filling pressures and pulmonary artery pressure, the impaired myocardial functional reserve is being considered another mechanism [35]. Overall, it is likely that both EAT and PAT independently and negatively impact LV diastolic function among the general population and patients with established CVD.

### Association of PAT with LV systolic function

In contrast to the solid evidence regarding the impact of EAT on LV diastolic function, an association of EAT or PAT with LV systolic function is still unclear. A series of recent studies using 2D/3D speckle tracking strain techniques for measuring LV systolic function in lieu of LV ejection fraction, although limited by small sample size, has indicated that EAT is associated with subclinical deterioration of LV systolic myocardial function, independent of general measures of obesity, such as BMI and waist/hip ratio [11, 36, 37]. Similarly, in patients with T2D, paracardial fat was also associated with subtle deterioration of LV systolic function assessed using 2D strain [4]. Our study extends these findings by showing that the regional effects of PAT (EAT plus paracardial fat) on LV systolic function have been found in the general population, independent of general obesity measures. However, because Eckel et al. showed that only secretary molecules derived from EAT of T2D patients directly impaired cardiomyocyte contractile function [38], more studies about the different roles of EAT and paracardial fat in patients with T2D are needed. Several new mechanisms have been proposed to explain the early influences of EAT on LV systolic function. NG et al. suggested that LV systolic dysfunction by excess EAT was modulated by a burden of interstitial myocardial fibrosis and increased intramyocardial TG content [36].

### Strengths and limitations

The major strengths of our study include the relatively large sample size from a population-based cohort, which reduced the potential for referral bias, and the use of a highly reproducible CT method for assessing PAT, which is the gold standard for fat quantification. Also, we assessed LV systolic and diastolic function using TDI measures, which can detect early subclinical deterioration in LV function. However, we did have limitations including use of a cross-sectional study design and evaluation of a single racial group. Thus, our findings may not be generalizable, and we cannot establish causality. Additionally, it should be mentioned that our study results may not be directly comparable with prior studies that measured only EAT instead of PAT because EAT and paracardial fat may contribute to the changes of LV structure and function in different ways. However, considering the fact that EAT and PAT are highly correlated (*r* = 0.92, *P* < 0.001) [21, 34] although there are slight differences between EAT and paracardial and their associations with metabolic risk factors [24], paracardial fat seems to have a similar relationship as seen between EAT and LV structure and function [21, 23, 34]. Another limitation includes the use of a wide range of HU scale, which might have overestimated PAT volume although there are no other tissues with these attenuation values within the inner thoracic cavity [39]. Finally, because the results of the present study were obtained from relatively healthy individuals free from CVD, further studies are needed to elucidate the role of PAT in patients with coronary artery disease or congestive heart failure, as obesity may confer mortality benefits in these subgroups [8].

## Conclusions

PAT accumulation is significantly associated with myocardial remodeling and subclinical impairment of LV systolic and diastolic function. Associations between PAT and cardiac structure and function are stronger than with other measures of general adiposity and thus represent a useful marker for obesity-associated cardiac changes.

## Data Availability

The data of this study are available from the corresponding authors on reasonable request and with permission of the Korean Centers for Disease Control and Prevention.

## Abbreviations

AF: Atrial fibrillation
ANOVA: Analysis of variance
BMI: Body mass index
CT: Computed tomography
CVD: Cardiovascular disease
DT: Deceleration time
EAT: Epicardial adipose tissue
HDL: High-density lipoprotein
LA: Left atrium
LV: Left ventricle
PAT: Pericardial adipose tissue
SD: Standard deviation
TDI: Tissue Doppler imaging
TG: Triglycerides
T2D: Type 2 diabetes
WC: Waist circumference
